# Non-cell autonomous mechanisms control mitochondrial gene dysregulation in polycystic ovary syndrome

**DOI:** 10.1530/JME-21-0212

**Published:** 2021-11-09

**Authors:** Alba Moreno-Asso, Ali Altıntaş, Luke C McIlvenna, Rhiannon K Patten, Javier Botella, Andrew J McAinch, Raymond J Rodgers, Romain Barrès, Nigel K Stepto

**Affiliations:** 1 Institute for Health and Sport (iHeS), Victoria University, Melbourne, Australia; 2 Australian Institute for Musculoskeletal Science (AIMSS), Victoria University, Melbourne, Australia; 3 Novo Nordisk Foundation Centre for Basic Metabolic Research, Faculty of Health and Medical Sciences, University of Copenhagen, Copenhagen, Denmark; 4 Discipline of Obstetrics and Gynaecology, School of Medicine, Robinson Research Institute, The University of Adelaide, Adelaide, South Australia, Australia

**Keywords:** polycystic ovary syndrome, skeletal muscle, myotubes, transcriptomics, mitochondria

## Abstract

Polycystic ovary syndrome (PCOS) is a common endocrine disorder associated with insulin resistance and impaired energy metabolism in skeletal muscle, the aetiology of which is currently unclear. Here, we mapped the gene expression profile of skeletal muscle from women with PCOS and determined if cultured primary myotubes retain the gene expression signature of PCOS *in vivo*. Transcriptomic analysis of *vastus lateralis* biopsies collected from PCOS women showed lower expression of genes associated with mitochondrial function, while the expression of genes associated with the extracellular matrix was higher compared to controls. Altered skeletal muscle mRNA expression of mitochondrial-associated genes in PCOS was associated with lower protein expression of mitochondrial complex II–V, but not complex I, with no difference in mitochondrial DNA content. Transcriptomic analysis of primary myotube cultures established from biopsies did not display any differentially expressed genes between controls and PCOS. Comparison of gene expression profiles in skeletal muscle biopsies and primary myotube cultures showed lower expression of mitochondrial and energy metabolism-related genes *in vitro*, irrespective of the group. Together, our results show that the altered mitochondrial-associated gene expression in skeletal muscle in PCOS is not preserved in cultured myotubes, indicating that the *in vivo* extracellular milieu, rather than genetic or epigenetic factors, may drive this alteration. Dysregulation of mitochondrial-associated genes in skeletal muscle by extracellular factors may contribute to the impaired energy metabolism associated with PCOS.

## Introduction

Polycystic ovary syndrome (PCOS) is the most common endocrine disorder in reproductive-aged women, affecting their metabolic, reproductive and mental health (Ehrmann 2005, Teede *et al.* 2010). The clinical hallmarks of PCOS include hyperandrogenism, ovulatory dysfunction and polycystic ovaries (Rotterdam ESHRE/ASRM-Sponsored PCOS Consensus Workshop Group 2004). At the pathophysiological level, PCOS has a strong metabolic component with insulin resistance being present in 38–95% of women with PCOS (Moghetti *et al.* 2013, Stepto *et al.* 2013, Tosi *et al.* 2017). Individuals with PCOS can present with a ~25% reduction in insulin sensitivity, independent of, but exacerbated by, obesity, when measured by euglycaemic–hyperinsulinaemic clamp (Cassar *et al.* 2016). Insulin resistance and compensatory hyperinsulinaemia are considered primary drivers of PCOS pathophysiology, contributing to hyperandrogenism and reproductive dysfunction (Dunaif *et al.* 1989, Teede *et al.* 2010). Insulin resistance in PCOS has been suggested to be mechanistically distinct from that of other metabolic disorders (Dunaif *et al.* 1989, Corbould 2008). However, there is a lack of understanding about the molecular mechanisms leading to insulin resistance in metabolic tissues (Stepto *et al.* 2019).

Skeletal muscle is a major contributor to insulin resistance accounting for 85% of post-prandial whole-body glucose disposal (DeFronzo *et al.* 1981). Numerous studies have attempted to elucidate the mechanisms involved in the development of skeletal muscle insulin resistance in PCOS, yet, the picture is unclear. Some evidence points towards downstream insulin signalling defects as causal factors leading to skeletal muscle insulin resistance, which occurs independently of obesity and which are distinct from those observed in type 2 diabetes (Dunaif *et al.* 1992, 2001, Corbould 2008, Højlund *et al.* 2008). Conversely, other studies have not been able to attribute any insulin signalling defects or decreased expression of genes of the insulin signalling pathway (Skov *et al.* 2007, Ciaraldi *et al.* 2009, Hansen *et al.* 2019, Stepto *et al.* 2020). Transcriptional investigations in skeletal muscle of insulin-resistant women with PCOS identified an association between insulin resistance and decreased mitochondrial oxidative phosphorylation (OXPHOS) transcripts (Skov *et al.* 2007), while other studies did not find such an association (Hutchison *et al.* 2012, Nilsson *et al.* 2018). Neither mitochondrial content markers, nor respiratory function, have been reported to differ between lean and overweight/obese insulin-resistant PCOS women compared to BMI-matched controls (Rabøl *et al.* 2011, Hutchison *et al.* 2012, Konopka *et al.* 2015). Thus, the contribution of mitochondrial dysfunction in insulin resistance in PCOS is unclear.

Primary myotube cultures allow to distinguish between cell-autonomous defects and adaptive mechanisms as a result of the influence of extracellular factors* in vivo*. A previous study showed that some of the insulin signalling defects observed *in vivo* are conserved in myotubes from insulin-resistant PCOS women when compared with controls, but this was not accompanied by a decrease in insulin sensitivity (Corbould *et al.* 2005). Conversely, several studies did not detect alterations in the insulin signalling pathway in myotubes from PCOS women (Ciaraldi *et al.* 2009, Eriksen *et al.* 2010). In contrast, the metabolic phenotype and signalling defects are more unanimously preserved in myotube cultures established from donors with type 2 diabetes (Gaster *et al.* 2002, Thingholm *et al.* 2011, Gaster 2019). The conflicting studies between PCOS and type 2 diabetes support the existence of distinct aetiological mechanisms of insulin resistance in these diseases.

The present study sought to gain further insight into the mechanisms leading to metabolic dysfunction in skeletal muscle in PCOS and to determine whether cell-autonomous factors are responsible for the dysregulation of skeletal muscle function in PCOS. Specifically, we investigated the transcriptomic profile of skeletal muscle and primary myotube cultures established from *vastuslateralis* biopsies from insulin-resistant women with PCOS compared to healthy controls. We hypothesized that skeletal muscle from women with PCOS would present with transcriptomic alterations and these would be mostly retained in primary myotube cultures.

## Materials and methods

### Study participants

In this study, eight overweight or obese insulin-resistant women with PCOS and seven lean healthy women were included. PCOS was diagnosed using the Rotterdam criteria, which requires any two of the following: (i) oligo- or anovulation; (ii) clinical and/or biochemical hyperandrogenism; (iii) polycystic ovaries on ultrasound and (iv) exclusion of other causes of hyperandrogenism or ovulatory dysfunction (Rotterdam ESHRE/ASRM-Sponsored PCOS Consensus Workshop Group 2004). Women with PCOS had their diagnosis confirmed by an endocrinologist. The healthy control group consisted of women without any features of PCOS. All women were premenopausal and aged between 18 and 40 years old. The exclusion criteria included menopause, pregnancy, smoking, type 1 or type 2 diabetes, uncontrolled hypertension (>160/100 mmHg), cardiovascular disease, renal impairment and malignancy and use of medications that interfere with endpoints (e.g. oral contraception, insulin sensitisers, anti-androgens, progestins, anti-hypertensives and lipid-lowering agents). Ethical approval was obtained from the Victoria University Human Research Ethics Committee (HRE17-232), and all participants provided written informed consent prior to participation in the study, as part of larger clinical trials (ACTRN12618000155291 and ACTRN12615000242527).

### Clinical and biochemical measures

All clinical measures were conducted after an overnight fast and in the early follicular phase of the menstrual cycle (days 1–7) for those participants with menstrual cycles. Participant’s body composition was assessed by dual energy X-ray absorptiometry (DXA) scan (iDXA GE Lunar Prodigy scanner) and performed by a licenced operator. A standard scanning protocol was used to ensure measurement reliability (Nana *et al.* 2015). BMI was calculated using height and weight measurements. Euglycaemic–hyperinsulinaemic clamps were performed to assess insulin sensitivity levels (DeFronzo *et al.* 1979). Insulin (NovoNordisk ActRapid) was infused at a constant rate (40 mU/min/m^2^) for approximately 120 min, with glucose infused at a variable rate to maintain a blood glucose level of 5 mmol/L. Blood glucose was assessed every 5 min using a glucose analyser (YSI 2300 STAT Plus). Glucose infusion rate (GIR) was calculated during steady state, defined as the last 30 min of the insulin-stimulated period and expressed as glucose (mg)/lean body mass (kg)/minute. Blood samples were collected during the fasted state from the antecubital vein. Plasma and serum were collected in appropriate blood tubes by centrifugation and stored at −80°C until samples were later batch-analysed. Plasma insulin levels were determined via radio-immuno assay kit from Millipore (Human Insulin-Specific RIA, HI-14K, Millipore), and serum anti-Müllerian hormone concentration was determined by ELISA (Ultra-Sensitive AMH/MIS ELISA, AL-105, Ansh Labs) at Victoria University. Total cholesterol, triglycerides, HDL-C, LDL-C, glycosylated haemoglobin (HbA1c), sex hormone binding globulin (SHBG), dihydrotestosterone, oestradiol (E2), androstenedione and testosterone were analysed in the accredited pathology laboratory at Monash Health, Melbourne, Australia, using standard protocols as previously described (Hiam *et al.* 2019).

### Collection of muscle biopsies and establishment of primary myotube cultures

Muscle biopsy samples from *vastuslateralis* were obtained from all participants following the modified Bergström technique (Bergstrom 1975, Shanely *et al.* 2014). Primary myotube cultures were successfully established using approximately 50 mg of muscle biopsy sample and following the method described previously (Agley *et al.* 2013). Isolated myoblasts were cultured in growth media (minimum essential medium alpha (α-MEM) with 10% v/v fetal bovine serum, 0.5% v/v penicillin–streptomycin and 0.5% v/v amphotericin B) until reaching 80% confluence. Cells were differentiated for 6 days in differentiation media (α-MEM with 2% v/v horse serum, 0.5% v/v penicillin–streptomycin and 0.5% v/v amphotericin B) until mature myotubes were formed.

### Measurement of glucose uptake in primary myotubes

Insulin-stimulated glucose uptake was performed using a radioactivity-based assay [^3^H]2-deoxy-d-glucose ([^3^H]2-DG) (Chanon *et al.* 2017). After an overnight incubation (16 h) in serum-free medium, differentiated myotubes were washed three times and pre-incubated with Kreb’s buffer (10 mM HEPES, 2.5 mM NaH_2_PO_4_, 150 mM NaCl, 5 mM KCl, 1.2 mM CaCl_2_, 1.2 mM MgSO_4_, 0.1% BSA) with or without 100 nM of insulin for 30 min at 37°C. A control condition with cytochalasin B was also included. Then, 10 μM 2-deoxy-d-glucose (2-DG) at 1 μCi/mL/well ([^3^H]2-DG) was added for exactly 15 min. Cells were then rinsed three times with cold PBS and lysed in 500 µL of 0.1 M NaOH. Four hundred microlitres of each lysate was transferred to scintillation vial while 100 µL was retained for quantification of total protein. Glucose uptake was determined using liquid scintillation counting on β-spectrometer (Perkin-Elmer). The unit of measurement was calculated in picomoles of [^3^H]2-DG taken up per minute per milligram was normalised to total protein.

### Determination of mitochondrial DNA copy number

Total DNA was extracted from skeletal muscle and myotubes using the Qiagen AllPrep DNA/RNA/miRNA universal kit (Qiagen). Mitochondrial DNA (mtDNA) copy number was determined in quadruplicates using multiplex quantitative PCR. This method allows for simultaneous amplification of a mitochondrial (ND1) and a nuclear (RNAseP) gene to verify the relative abundance of mtDNA over nuclear DNA (ncDNA) (Krishnan *et al.* 2007). RNAseP was assessed by using the RNAseP assay kit (Thermo Fisher Scientific), and the primer and probe sequences to amplify ND1 (IDT) are as follows: forward primer (300 nM), 5’CCCTAAAACCCGCCACATCT3’; reverse primer (300 nM): 5’GAGCGATGGTGAGAGCTAAGGT3’; and probe (100 nM): 5’FAMCCATCACCCTCTACATCACCGCCC-TAMRA3’. Taqman Universal Mastermix (Thermo Fisher Scientific) was used and the assay was run on a QuantStudio™ 7 Flex Real-Time PCR System (Applied Biosystems). The average coefficient of variation for both mtDNA and ncDNA threshold cycles (Cts) was 1.1%. Data were manually curated and in the case of a s.d. of more than 0.3 for the Cts of the quadruplicates, the outlier value was suppressed from the analysis. Results were expressed as relative mtDNA/ncDNA ratio calculated by the ΔΔCt method using the mean of the muscle from healthy controls as reference sample (Quiros *et al.* 2017).

### RNA sequencing

Total RNA was extracted from skeletal muscle biopsies of eight women with PCOS and six healthy women, and from primary myotubes from five women with PCOS and six healthy women using the Qiagen AllPrep DNA/RNA/miRNA universal kit (Qiagen). Agilent RNA 600 Nano kit and Bioanalyzer instrument (Agilent Technologies) was used to assess the quality of the total RNA samples (500 ng). Sequencing libraries were prepared according to the TruSeq stranded total RNA with the Ribo-Zero Gold protocol (Illumina), as previously described (Hiam *et al.* 2019). Quantification of libraries was performed using the Qubit dsDNA HS assay kit (Invitrogen) to ensure optimum cluster densities. Quality control for base pair size and purity was assessed using an Agilent high-sensitivity DNA chip and Bioanalyzer instrument (Agilent Technologies). Each library was diluted to 1 nM before being pooled and sequenced on the NovaSeq6000 (Illumina).

### Bioinformatic analysis of RNA sequencing data

Sequencing reads (*n* ≈ 35.3 M) from FASTQ files were aligned using STAR (v2.7.2b) aligner with Ensembl human annotation (GRCh38, release 98), and gene features were counted using *featureCounts* from subread (v1.6.2) package resulting in 26.5 M and 22 M reads on average, respectively (Supplementary Fig. 1, see section on supplementary materials given at the end of this article). One library (sample: C_M_pre_2) was excluded from downstream analysis due to low read number (*n* = 17,549). The lowly expressed genes were discarded from downstream analysis using *filterByExpr* function from edgeR package (v3.28.1) resulting in 17,689 genes. To calculate the differentially expressed genes, a generalized linear model was fit by edgeR’s (v3.32.1) *voomLmFit* function while blocking for participants. Genes with false discovery rate (FDR) < 0.05 were considered as differentially expressed. Multidimensional scaling (MDS) plots were created by using batch (participants)-corrected logCPM values. All sequencing data are available under Gene Expression Omnibus data repository.

### Pathway enrichment analysis

Pathway enrichment analysis was performed using the gene set enrichment analysis (GSEA) and over-representation analysis (ORA) methods as indicated. Both GSEA and ORA were performed by using clusterProfiler (v3.14.3) and Reactome database (ReactomePA R package, v1.30.10). The log_2_-fold-changes (logFC) calculated during the differential expression analysis were ranked and permuted 1 million times for GSEA. The ontologies with less than 10 and more than 500 genes were discarded. Remaining ontologies with *q*-value < 0.05 were considered as enriched.

### Cluster analysis of expression patterns

Batch (participant)-corrected gene expression values, logCPMs, were used for clustering analysis for each experimental group, which are defined as a combination of cell type (myotube, muscle) and disease (control, PCOS). The clustering analysis was performed using ‘clust’ algorithm (Abu-Jamous & Kelly 2018), which resulted in 10 distinct clusters. Gene enrichment analysis using Reactome database and ORA method was performed for each cluster with the same parameters mentioned above.

### Quantitative reverse transcriptase PCR

Extracted mRNA of skeletal muscle and myotube samples from eight women with PCOS and six healthy women were reverse transcribed using Bio-Rad iScript™ RT Supermix (Bio-Rad Laboratories) and a Thermocycler (Bio-Rad Laboratories). Quantitative reverse transcriptase PCR (qRT-PCR) reactions were performed in triplicate using SsoAdvanced Universal SYBR Green Supermix (Bio-Rad Laboratories) and amplified by QuantStudio™ 7 Flex Real-Time PCR System (Applied Biosystems). Specific pairs of qRT-PCR primers for nine selected genes were used (Table 1). Gene expression results were represented as 2^−ΔCt^ normalised to the geometric mean of the three most stable housekeeping genes (*PPIA* (*Cyclophilin*), *B2M*, *TBP*) out of five analysed (*TBP*, *ACTB*, *Cyclophilin*, *B2M*, *GAPDH*), selected using RefFinder (https://www.heartcure.com.au/reffinder/).

**Table 1:** qRT-PCR primer sequences.

Gene symbol	Primer sequence 5’– 3’	Accession number
*RPL39*	F: TGTTCTTGACTCCGCTGCTC R: TCTCCTTTTGGAGTTGTACCTGA	NM_001000.4
*MYL6B*	F: AACCCCAAGAGTGATGAGCTG R: CACACGAAACCCCTCCAAGT	NM_001199629.2
*PRKAG3*	F: AGTCCTCAATCCCAAGCCAC R: AGGGCTGAAGAAGCCTGAATC	NM_017431.3
*LPL*	F: GATCCATGGCTGGACGGTAA R: GACAGCCAGTCCACCACAAT	NM_000237.2
*NDUFA9*	F: CGCATGGGGTCACAGGTAAT R: CTCGCGTCCCATTCCAGAAA	NM_005002.5
*SDHB*	F: AAATGTGGCCCCATGGTATTG R: AGAGCCACAGATGCCTTCTCTG	NM_003000.3
*UQCRC2*	F: GCAGTGACCGTGTGTCAGAA R: AGGGAATAAAATCTCGAGAAAGAGC	NM_003366.4
*COX4A1*	F: GAGCAATTTCCACCTCTGC R: CAGGAGGCCTTCTCCTTCTC	NM_001861.6
*ATP5PD*	F: CCTCACCTCCAGGTTGGC R: GCACAAGATTTCACCTTCTTCTCA	NM_001003785.2
*MRPS7*	F: GCAGCTTCCAGGGCTAACT R: CCTCCACTGGCTTGCGATA	NM_015971.4
*MRPL41*	F: GACCGAATGAGCAAGTGGAC R: CTCCTTGATCTGCACGAACCT	NM_032477.3
*ACTB*	F: GAGCACAGAGCCTCGCCTTT R: TCATCATCCATGGTGAGCTGGC	NM_001101.3
*PPIA*	F: GTCAACCCCACCGTGTTCTTC R: TTTCTGCTGTCTTTGGGACCTTG	NM_021130.4
*B2M*	F: TGCTGTCTCCATGTTTGATGTATCT R: TCTCTGCTCCCCACCTCTAAGT	NM_004048.2
*GAPDH*	F: AATCCCATCACCATCTTCCA R: TGGACTCCACGACGTACTCA	NM_001289746.1
*TBP*	F: CAGTGACCCAGCAGCATCACT R: AGGCCAAGCCCTGAGCGTAA	NM_003194.4

F, forward primer; R, reverse primer.

### Protein immunoblotting

Protein content was detected by Western blot using skeletal muscle samples from eight women with PCOS and six healthy controls, and six myotube cultures from women with PCOS and six from healthy controls (Supplementary Fig. 2 and Supplementary document 1). For each protein of interest, a signal linearity test was conducted to determine the ideal loading amount. Muscle lysates were diluted in 4× Laemmli buffer (0.25 M Tris, 4% SDS, 20% glycerol, 0.015% bromophenol blue and 10% 2‐mercaptoethanol) and were then loaded in equal amounts (10–20 μg) on a Criterion™ 4–20% TGX Stain‐Free™ Precast Gels (Bio‐Rad Laboratories). Samples were then separated by electrophoresis for 1.5–2.5 h at 100 V before wet transferral onto low-fluorescence PVDF membranes using a Turbo Transfer system (Bio-Rad Laboratories). Membranes were blocked at room temperature for 1 h using 3% skim milk in Tris buffer saline (TBS) 0.1% tween-20 (TBS-T). After 3 × 5-min washes in TBS-T, membranes were incubated overnight at 4°C with gentle agitation in primary antibody solutions (1:1000 antibody in 3% BSA, plus 0.02% sodium azide). The following antibodies from Abcam were used: UQCRC2 (ab14745), COX IV (ab14744), SDHA (ab14715), NDUFA9 (ab14713), ATP5A (ab14748), MRPS7 (ab138088) and MRPL41 (ab121821). Membranes were next washed 3 × 5 min in TBS-T and subsequently incubated under gentle agitation at room temperature with the appropriate host species-specific secondary antibody for 90 min in 3% skim milk in TBS-T. Membranes were washed again for 3 × 5-min in TBS-T before being immersed under gentle agitation at room temperature in Clarity ECL detection substrate (Bio-rad Laboratories) or SuperSignal West Femto (ThermoFisher Scientific). Protein bands were visualised using a Bio-Rad ChemiDoc imaging system, and band densities were determined using Bio-Rad ImageLab software (Bio-Rad Laboratories).

### Statistical analysis

Clinical characteristics of groups were compared with two-tailed unpaired Student’s *t*-test. Statistical analyses for glucose uptake, gene expression, protein abundance and mtDNA copy number were performed by two-way repeated measures of ANOVA with Bonferroni adjustment for multiple comparisons using GraphPad Prism software version 8.2.1 (GraphPad Software Inc.). The distributions of the data were tested using Shapiro–Wilk test; when data were not normally distributed, normality was achieved by log transformation. All data are reported as mean ± s.d., and statistical significance was declared when *P*_adj_ < 0.05.

## Results

### Endocrine and metabolic characteristics of the subjects

Women with PCOS were overweight with increased BMI, fat mass percentage and reduced lean mass percentage compared to healthy controls (*P* < 0.001) (Table 2). Women with PCOS also had significantly lower levels of SHBG, higher levels of total and free testosterone, androstenedione, low-density lipoprotein, LDL:HDL ratio, and fasting glucose than healthy control women (*P* < 0.05) (Table 2). GIR in women with PCOS was 49.5% lower than controls (*P* < 0.001) as measured by euglycaemic–hyperinsulinaemic clamp (Table 2). These clinical characteristics are in line with the previously reported phenotypical differences observed in insulin-resistant women with PCOS when compared to controls (Skov *et al.* 2007, Stepto *et al.* 2013).

**Table 2:** Clinical characteristics.

	Controls (*n* = 7)	PCOS (*n* = 8)
Age	25.7 ± 5.7	28.3 ± 2.5
Body composition		
Weight (kg)	64.5 ± 12.9	97.8 ± 13.3^a^
BMI (kg/m^2^)	22.2 ± 2.6	35.7 ± 5.7^a^
Body fat (%)	29.3 ± 4.7	49.2 ± 5.4^a^
Lean mass (%)	70.6 ± 8.4	48.4 ± 4.9^a^
Glucose homeostasis		
HbA1c (%)	5.13 ± 0.15	5.16 ± 0.04
Fasting insulin (µIU/mL)	9.98 ± 2.44	14.64 ± 6.30
Fasting glucose (mmol/L)	4.44 ± 0.34	5.01 ± 0.29^b^
Insulin sensitivity		
GIR (mg/lbm kg/min)	16.36 ± 4.76	7.23 ± 3.34^a^
Hormonal status		
Total testosterone (nmol/L)	0.92 ± 0.31	1.61 ± 0.65^b^
Free testosterone (pmol/L)	13.54 ± 5.99	37.07 ± 13.98^b^
SHBG (nmol/L)	54.01 ± 24.51	26.71 ± 10.76^b^
Dihydrotestosterone (nmol/L)	0.33 ± 0.18	0.32 ± 0.13
Androstenedione (nmol/L)	3.23 ± 0.85	4.83 ± 1.17^b^
Estradiol (pmol/L)	179.96 ± 186.40	178.73 ± 115.28
Anti-Müllerian hormone (pmol/L)	76.73 ± 40.30	74.11 ± 28.09
Lipids		
Cholesterol (mmol/L)	3.87 ± 0.67	4.70 ± 0.80
Triglycerides (mmol/L)	0.64 ± 0.09	1.04 ± 0.42
High-density lipoprotein (mmol/L)	1.57 ± 0.36	1.34 ± 0.25
Low-density lipoprotein (mmol/L)	2.02 ± 0.57	2.90 ± 0.68^b^
LDL:HDL ratio	1.39 ± 0.54	2.21 ± 0.51^b^

Data presented as mean ± s.d.

^a^*P <* 0.001 vs controls*. ^b^P* < 0.05 vs controls.

### Altered muscle expression of genes controlling mitochondrial function in PCOS

**Figure 1:**
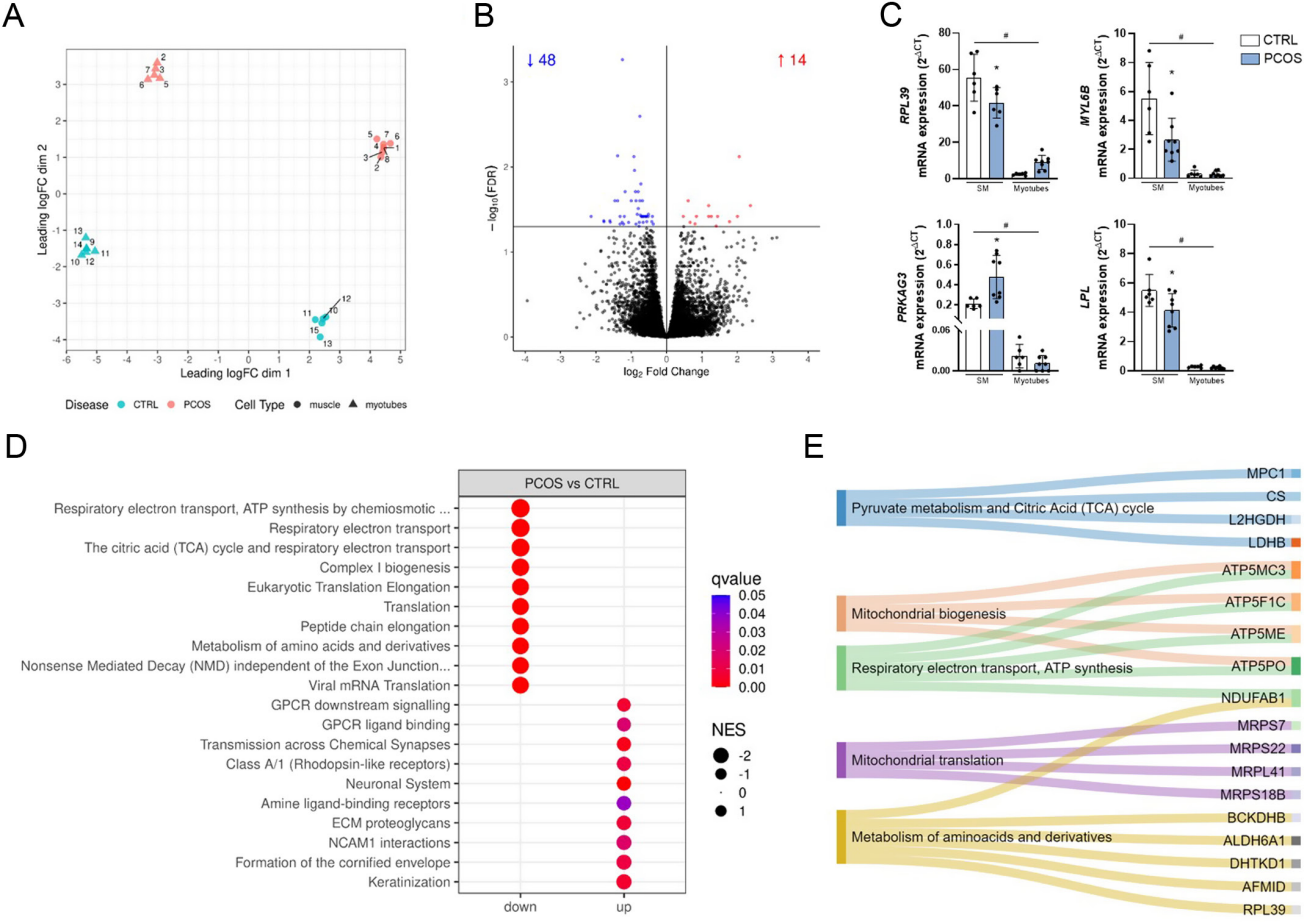
Gene expression analysis of skeletal muscle. (A) Multidimensional scaling (MDS) plot of RNA-seq data from skeletal muscle (SM) (muscle) and primary myotube cultures (myotubes) from women with PCOS (PCOS) and healthy control women (CTRL). (B) Volcano plot of all transcripts detected between SM of women with PCOS and healthy controls, with significantly upregulated genes (red) and downregulated genes (blue) highlighted. (C) mRNA abundance of selected significantly differentially expressed genes in RNA-seq data from SM and myotubes from healthy controls (CTRL, *n*  = 6; in white) and PCOS women (PCOS, *n*  = 8; in blue). Gene expression is expressed as 2^−∆CT^ and represented as mean ± s.d. *P_adj_ < 0.05 vs skeletal muscle from healthy controls; ^#^*P*_adj_ < 0.05 myotubes vs skeletal muscle of each group, respectively. (D) Top 10 upregulated and downregulated Reactome pathways from GSEA in skeletal muscle (*q*-value < 0.05). (E) Sankey diagram of Reactome pathways and associated genes from over-representation analysis (ORA) of differentially downregulated genes.

To determine the gene expression profile of skeletal muscle in PCOS, we performed transcriptomic analysis by RNA sequencing (RNA-seq) of skeletal muscle biopsies from women with PCOS and healthy controls. MDS plots of RNA-seq data showed a marked separation by group (PCOS vs controls) and between skeletal muscle and primary myotubes, and did not suggest the presence of outliers (Fig. 1A). We did not detect any effect of age (Supplementary Fig. 3A), BMI (Supplementary Fig. 3B) or any other confounding clinical variables on the PCOS-specific gene expression profile. In total, we detected a total of 17,690 transcripts, of which 62 genes were differentially expressed (14 upregulated and 48 downregulated; FDR < 0.05) in skeletal muscle of women with PCOS (Fig. 1B and Supplementary List 1). Differential expression of a selection of genes *RPL39*, *MYL6B*, *LPL* and *PRKAG3* was validated by qRT-PCR (Fig. 1C). GSEA using the Reactome pathway database (Supplementary List 2) showed that genes associated with mitochondrial function and protein translation are specifically downregulated in the skeletal muscle of women with PCOS compared to controls (Fig. 1D). The main mitochondrial functions that were downregulated included the citric acid (TCA) cycle, mitochondrial biogenesis, ATP synthesis, respiratory electron chain, mitochondrial protein import, and mitochondrial protein translation. Conversely, upregulated pathways were related to keratinization, cell adhesion via NCAM1 interactions, neurotransmitters and chemical synapses, extracellular matrix (ECM) proteoglycans, and GPCR signalling (Fig. 1D). Similar over-represented Reactome pathways were obtained for the restricted list of 48 significantly downregulated genes (Supplementary List 3), with a marked over-representation of genes involved in mitochondrial pathways such as pyruvate metabolism and citric acid (TCA) cycle, mitochondrial biogenesis, respiratory electron chain and ATP synthesis, mitochondrial translation, and metabolism of amino acids and derivatives (Fig. 1E). Thus, compared to control group, the skeletal muscle of women with PCOS is characterised by lower expression levels of genes involved in several mitochondrial functions and higher expression of transcripts involved in ECM components.

### The skeletal muscle gene expression profile in PCOS is lost in cultured myotubes

**Figure 2:**
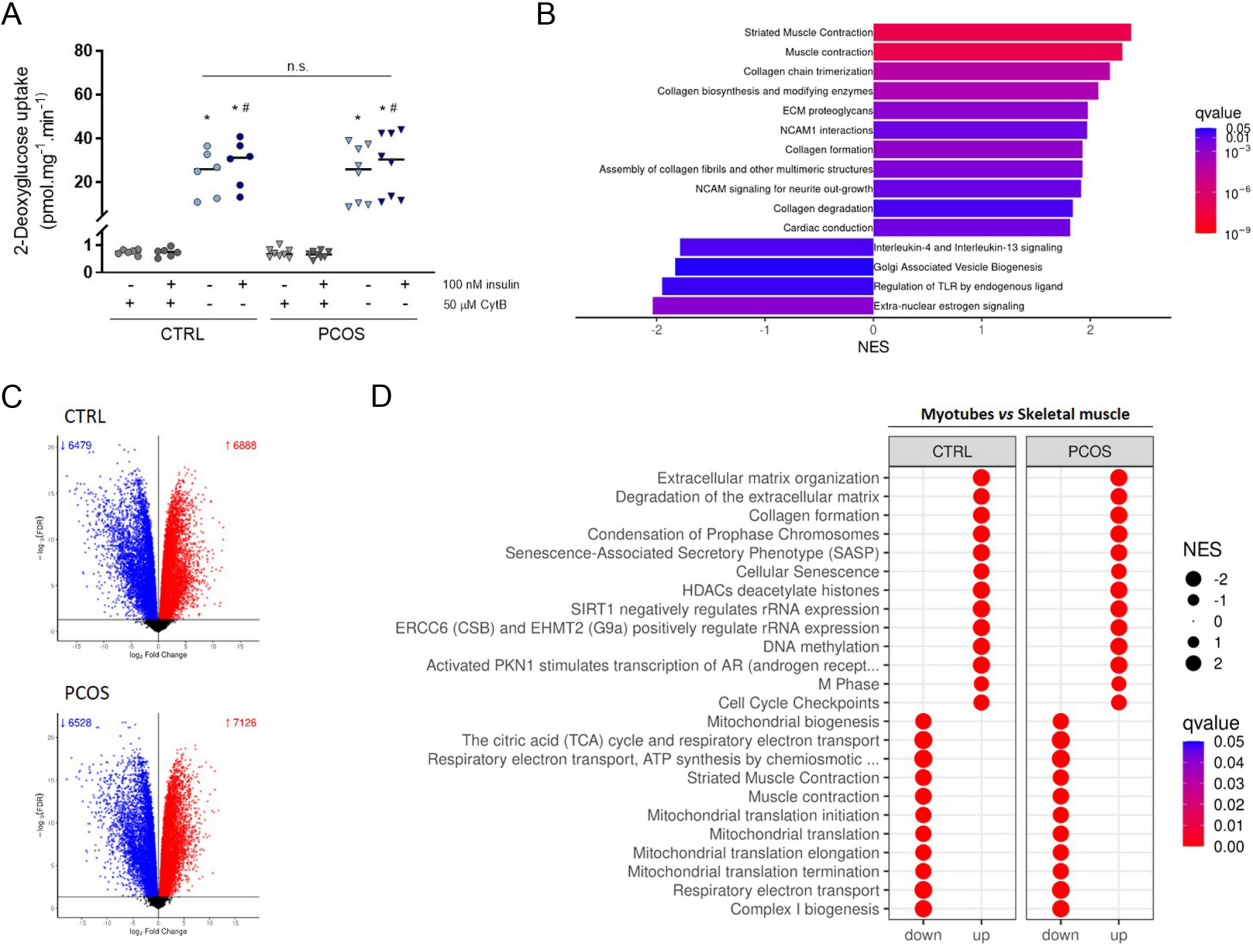
Glucose transport and gene expression analysis of primary myotubes. (A) Glucose uptake of primary myotubes from healthy control women (CTRL, *n*  = 6) and women with PCOS (PCOS, *n*  = 8) showing non-insulin and insulin-stimulated data with and without cytochalasin B (CytB). Line represents the mean value for each group. *P_adj_ < 0.05 vs CytB; ^#^*P_adj_* < 0.05 vs basal (non-insulin stimulated without cytB); n.s. means non-significant difference between PCOS and CTRL. (B) Bar plot of top Reactome pathways from GSEA in myotubes from women with PCOS compared to those from controls (*q*-value < 0.05). (C) Volcano plots of all transcripts detected between myotubes and skeletal muscle from healthy controls (CTRL) or PCOS women (PCOS), with significantly (FDR < 0.05) upregulated genes (red) and downregulated (blue) highlighted. (D) Top 10 upregulated (up) and downregulated (down) Reactome pathways (*q*-value < 0.05) from GSEA in myotubes compared to skeletal muscle for both healthy controls (CTRL) and PCOS women (PCOS), respectively.

We next aimed to determine whether the altered skeletal muscle transcriptomic signature in PCOS is retained *in vitro*. To address this, we characterised the gene expression profile of primary myotube cultures. Here also, MDS plots of gene expression data show a marked separation between PCOS and controls, as well as between muscle and primary myotubes (Fig. 1A). We investigated the differences in gene expression between PCOS and controls in primary myotube and found no differentially expressed transcripts (FDR > 0.05) (Supplementary List 4). The lack of differential expression was accompanied by no difference in glucose uptake at basal or insulin-stimulated conditions in cultured myotubes from women with PCOS compared to healthy controls (Fig. 2A). While no significant genes were differentially expressed, GSEA of Reactome pathways analysis returned an enrichment of genes related to an upregulation of muscle contraction, ECM organisation and extracellular signalling in PCOS myotubes and lower representation of intracellular processes including signalling regulation and Golgi vesicles biogenesis (Fig. 2B and Supplementary List 5). Thus, our results show that, contrary to skeletal muscle tissue, there is no evident gene expression difference in primary muscle cell cultures from women with PCOS compared to controls, despite a significant enrichment on muscle physiology, ECM components and signalling regulation in the overall gene expression pattern. This suggests that alteration of the skeletal muscle gene expression profile in PCOS may be driven by the extracellular milieu *in situ*, rather than genetic or epigenetic factors.

### Expression of mitochondria-related genes is altered in primary myotubes

**Figure 3:**
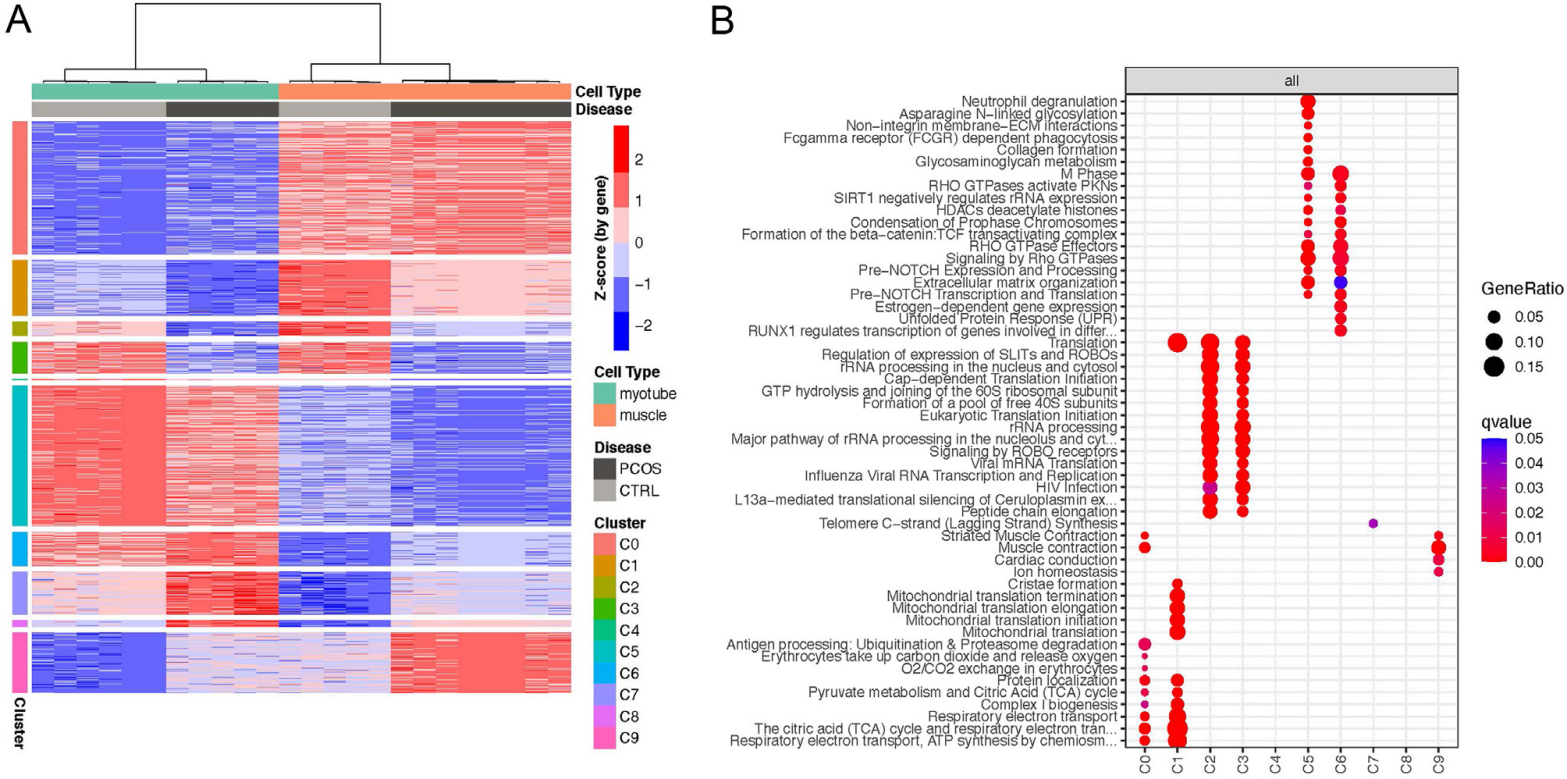
Cluster analysis of gene expression. (A) Heatmap representing cluster analysis results of upregulated (red) and downregulated (blue) genes in myotubes (top green bar) and skeletal muscle (top orange bar) samples from both healthy controls (CTRL; top light grey bar) and PCOS women (PCOS; top dark grey bar). X-axis: Clusters calculated by “clust” algorithm, y-axis: hierarchical clustering of samples using Pearson distance and ‘ward.D2’ algorithm. (B) Over-representation analysis (ORA) of Reactome pathways (*q*-value < 0.05) for each of the gene clusters.

We next compared gene expression between primary myotubes and skeletal muscle and found a total of 13,654 differentially expressed genes in PCOS (7126 upregulated and 6528 downregulated; FDR < 0.05), whereas 13,367 genes (6888 upregulated and 6479 downregulated; FDR < 0.05) were differentially expressed in controls (Fig. 2C and Supplementary List 6). To get functional insight into the difference in gene expression between muscle tissue and cultured primary myotubes, we performed GSEA (Supplementary List 7). From both PCOS and controls, the top downregulated pathways were associated to mitochondria: mitochondrial biogenesis, respiratory electron transport, the citric acid (TCA) cycle, mitochondrial translation, and muscle contraction (Fig. 2D). Contrary, the upregulated genes in both groups were enriched in pathways involved in ECM organisation, chromatin structure, cellular senescence, and transcriptional regulation (Fig. 2D). These findings were strengthened by the cluster analysis, which identifies groups of genes with the same expression pattern. Genes in clusters C0 and C1 were downregulated in primary myotubes compared to skeletal muscle in both PCOS and control women (Fig. 3A). These gene clusters were enriched in pathways associated to mitochondria (Fig. 3B), consistent with the findings from the abovementioned GSEA. Conversely, the genes in clusters C5 and C6 had higher expression in myotubes compared to skeletal muscle (Fig. 3A) and were identified to be mainly involved in ECM components and organisation (Fig. 3B). Altogether, these data show that genes involved in mitochondrial function and energy metabolism are altered in primary muscle cell cultures compared to skeletal muscle tissue, supporting that these genes are regulated by extracellular factors *in vivo*.

### Dysregulation of mitochondria-related genes is observed at the protein level and is independent of mtDNA content

**Figure 4:**
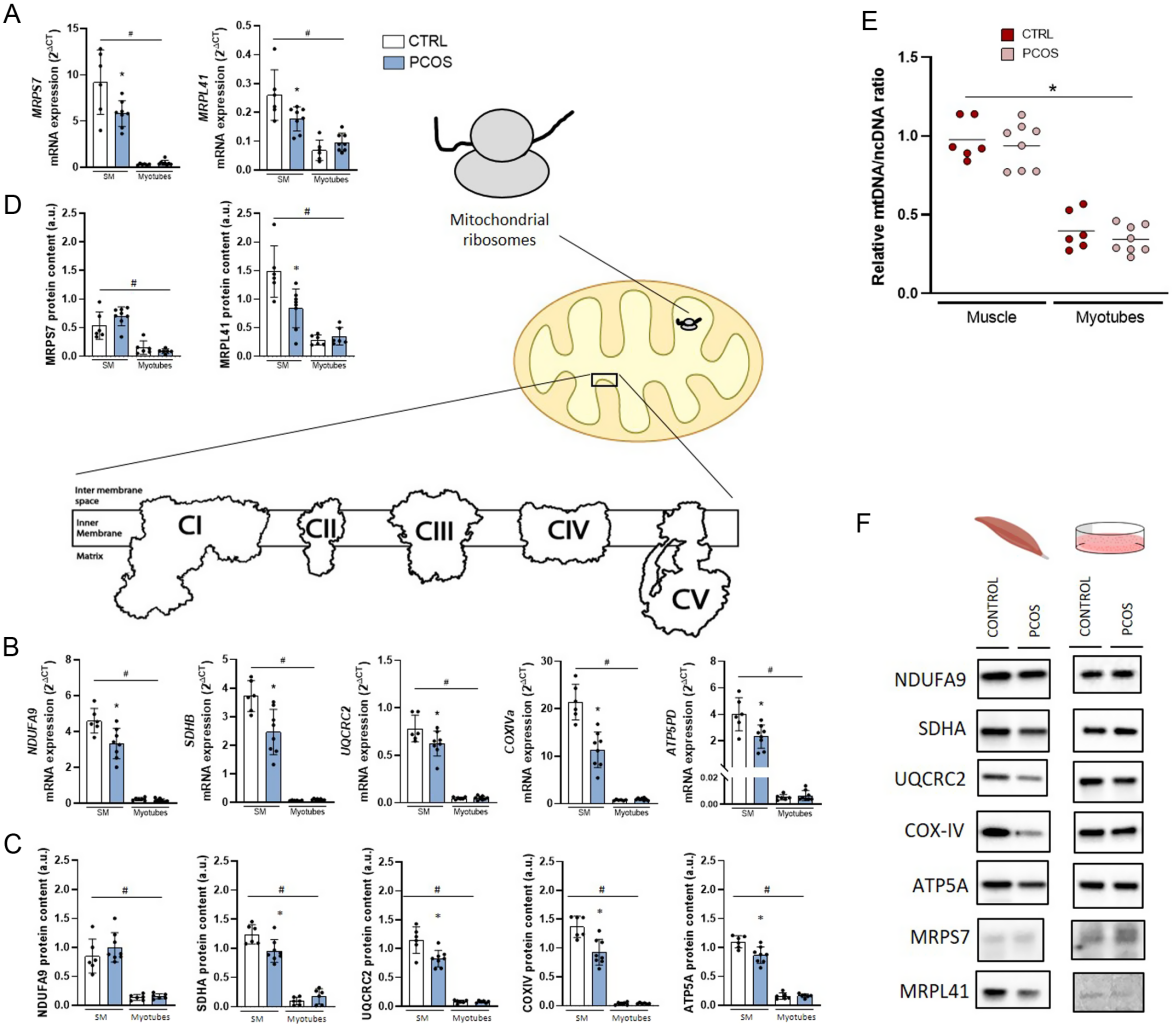
Gene expression and protein abundance of mitochondrial-associated genes and mtDNA copy number in skeletal muscle (SM) and primary myotubes. (A and B) mRNA of mitochondrial OXPHOS complexes I–V (*NDUFA9, SDHB, UQCRC2, COX4a1* and *ATP5PD*) and mitochondrial ribosomes *MRPS7* and *MRPL41* in SM and primary myotubes. Gene expression is expressed as 2^−∆CT^ and represented in bar plots showing individual data. (C and D) Protein abundance of mitochondrial OXPHOS complexes I–V (NDUFA9, SDHA, UQCRC2, COX4a1 and ATP5A) and mitochondrial ribosomes MRPS7 and MRPL41 is expressed in arbitrary units (a.u) and represented in bar plots showing individual data. (E) Relative mtDNA/ncDNA ratio expressed as 2^−^^∆∆CT^ for both SM and myotubes samples from healthy control (CTRL, *n*  = 6) and PCOS (PCOS, *n*  = 8) women. Line represents the mean value for each group. *P < 0.05 between SM and myotubes. (F) Representative immunoblots for each of the targets for SM and primary myotubes samples. Healthy controls (CTRL, *n*  =6) are represented using white bars and women with PCOS (PCOS, *n*  =8) in blue. Data presented as mean ± s.d.. *P_adj_ < 0.05 vs skeletal muscle from healthy controls; ^#^*P*_adj_ < 0.05 myotubes vs skeletal muscle.

Given the decreased transcript levels of mitochondria-related genes in skeletal muscle of women with PCOS compared to controls, as well as in primary myotubes regardless of the group, we next sought to investigate the expression levels of mitochondrial-associated proteins. We measured the expression of a selected representative gene and protein from each of the five respiratory complexes (I–V) (NDUFA9, *SDHB*/SDHA, UQCRC2, COX4a1 and *ATP5PD*/ATP5A) involved in OXPHOS and two mitochondrial ribosome proteins (MRPS7, MRPL41). Confirming results from the pathway enrichment analysis, qRT-PCR results showed a significant lower expression of the respiratory complexes genes *NDUFA9* (*P* = 0.001), *SDHB* (*P* < 0.001), *UQCRC2* (*P* = 0.024), *COX4a1* (*P* < 0.001) and *ATP5PD* (*P* = 0.002) and the mitochondrial ribosome genes *MRPL41* (*P* = 0.031) and *MRPS7* (*P* = 0.008) in skeletal muscle of women with PCOS (Fig. 4A, B and Supplementary Fig. 2). We also confirmed a significant lower expression of all these genes in primary myotubes compared to skeletal muscle in both groups (*P* < 0.001) and no differences in primary myotubes between PCOS and controls (Fig. 4A and B).

Protein expression of four out of the five OXPHOS complexes was found to be downregulated in the skeletal muscle of women with PCOS (Fig. 4C). Complex I (NDUFA9) was not significantly decreased (*P* = 0.32), while complex II (SDHA) showed a reduction of 29% (*P* = 0.014), complex III (UQCRC2) was decreased by 39% (*P* = 0.007), complex IV (COX4a1) was reduced by 47% (*P* = 0.002), complex V (ATP5A) was decreased by 26% (*P* = 0.006) (Fig. 4C). The mitochondrial ribosome MRPL41 was lower by 76% (*P* = 0.01), while MRPS7 was not significantly lower (*P* = 0.15) (Fig. 4D). In line with the transcriptomic findings, a marked reduction in mitochondrial ribosomes (*P* < 0.05) and OXPHOS complexes protein content (*P* < 0.001) was detected in primary myotubes compared to skeletal muscle, and no significant differences were observed between myotubes from control and PCOS women (Fig. 4A and  B). These results confirm that transcriptomic differences in PCOS skeletal muscle of OXPHOS complexes (II–V) and *MRPL41* are translated into differences in protein abundance. Notably, mtDNA content was not distinct between PCOS and healthy control women in skeletal muscle and myotubes. However, there was a significant reduction in mtDNA content in myotubes when compared to skeletal muscle (*P* < 0.05), in accordance with the GSEA data (Fig. 4E). Altogether, our results indicate that lower transcriptomic expression of mitochondria-related genes in skeletal muscle from women with PCOS is associated to a dysregulation at the protein level and is independent of mtDNA content.

## Discussion

Here, we used a transcriptomic approach to investigate the possible contribution of cell-autonomous factors in the gene expression profile of skeletal muscle from women with PCOS. We found that mitochondrial-associated gene pathways represent the main gene expression difference in skeletal muscle of insulin-resistant women with PCOS compared to healthy controls. We show that alteration of mitochondria-related genes is lost in primary muscle cell cultures, indicating that extracellular factors present in the *in vivo* milieu may be responsible for the gene expression reprogramming of mitochondrial function associated genes in PCOS.

To the best of our knowledge, this study is the first whole-transcriptome analysis performed in skeletal muscle and primary myotubes from women with PCOS. Two studies have previously investigated the skeletal muscle transcriptome in PCOS using array-based gene expression analysis (Skov *et al.* 2007, Nilsson *et al.* 2018). One of the studies, which included overweight insulin-resistant women with PCOS and BMI-matched controls, reported a decreased expression in OXPHOS genes in skeletal muscle in PCOS, in accordance with our findings (Skov *et al.* 2007). Conversely, Nilsson *et al.* identified 85 differentially expressed transcripts in skeletal muscle from women with PCOS (Nilsson *et al.* 2018), but contrary to the findings from Skov *et al.* (2007), none of these genes were differentially expressed in our study nor were they associated with mitochondrial pathways. These discrepancies may be explained by the difference in the metabolic characteristics of the participants as Nilsson *et al.* did not analyse insulin-resistant subjects, with both groups having similar HOMA-IR, HOMA-B or glucose disposal rate (Nilsson *et al.* 2018). In our study, BMI was not matched between the PCOS and control groups, thus, obesity may represent a confounding factor. However, we did not detect any effect of BMI within groups, suggesting that obesity might not have a major effect on the PCOS-specific gene expression profile in skeletal muscle. It is noteworthy to highlight that despite the limitation of not having BMI- and even age-matched participants, our transcriptomic results are similar to those of the aforementioned study which compared overweight/obese insulin-resistant PCOS women with age- and BMI-matched controls (Skov *et al.* 2007). The dysregulation of genes involved in mitochondrial function is therefore likely to be specific of PCOS insulin resistance itself rather than a characteristic solely driven by obesity. Moreover, a follow-up study further supported the link between dysregulation of mitochondrial-associated genes and insulin resistant in PCOS by showing that pioglitazone treatment improves skeletal muscle insulin sensitivity by upregulation of genes involved in mitochondrial OXPHOS and ribosomal protein biosynthesis in women with PCOS (Skov *et al.* 2008).

Our study identified transcriptional changes in OXPHOS genes which are accompanied by a substantial decrease of complex II–V proteins, but not complex I. Despite this decreased expression of OXPHOS complexes may suggest that mitochondrial content may be reduced in PCOS women, we did not observe any mtDNA content difference in skeletal muscle of women with PCOS. While we did not assess any other biomarkers of mitochondrial content, our results are consistent with previous studies, which failed to detect mitochondrial content alterations in skeletal muscle from both overweight/obese and lean insulin-resistant women with PCOS (Rabøl *et al.* 2011, Hutchison *et al.* 2012, Konopka *et al.* 2015). However, comprehensive studies using electron microscopy imaging, the gold-standard mitochondrial content marker, as done in obesity and type 2 diabetes (Kelley *et al.* 2002, Chomentowski *et al.* 2011), are needed to conclude whether mitochondrial abnormalities in skeletal muscle from PCOS women are due to differences in volume density, morphology or structure.

We detected that pathways associated with ECM organisation, cell adhesion and extracellular signalling molecules were enriched in skeletal muscle from women with PCOS. Dysregulated transforming growth factor-beta (TGFB) signalling and increased production and deposition of collagen has been previously described in the ovaries of women with PCOS (Raja-Khan *et al.* 2014, Bastian *et al.* 2016). Deposition of ECM components has been proposed to be involved in the development of PCOS and to contribute to metabolic abnormalities in other tissues (Raja-Khan *et al.* 2014). In this regard, and in agreement with our observations, a recent study by our group identified altered gene expression of TGFB ligands and components of the ECM, including collagens, in skeletal muscle of insulin-resistant overweight PCOS compared with BMI-matched control women (Stepto *et al.* 2020). Together, these observations support the existence of a PCOS-specific ECM signature in skeletal muscle and propose a potential role for these extracellular alterations in the metabolic dysfunction of this tissue.

The main objective of our work was to investigate whether the skeletal muscle gene expression signature of insulin-resistant PCOS women is preserved in cultured primary muscle cells. Evidence from other metabolic disorders suggests that primary myotubes derived from women with PCOS may retain the *in vivo* metabolic characteristics of their donor; however, current studies in PCOS are inconclusive (Corbould *et al.* 2005, Ciaraldi *et al.* 2009, Eriksen *et al.* 2010). In our study, primary myotube cultures established from women with PCOS did not retain insulin resistance, as shown by insulin-stimulated glucose transport, and did not show altered expression of genes related to mitochondrial function. This was also confirmed at protein level for the mitochondrial ribosomes and OXPHOS complexes, with no detectable differences in cultured myotubes from women with PCOS when compared to controls. These results are strengthened by a previous study that also failed to detect any alterations in mitochondrial function or content in myotubes established from insulin-resistant women with PCOS (Eriksen *et al.* 2011). This loss of *in vivo* characteristics in primary myotubes suggests that factors present in the extracellular milieu *in vivo* may be regulating the expression of mitochondrial function associated genes in skeletal muscle of women with PCOS. Remarkably, these findings are in contrast with evidence in type 2 diabetes and obesity, where several defects in insulin signalling, insulin‐stimulated glucose metabolism and an impairment of lipid oxidation, ATP synthesis and OXPHOS have been described to be conserved in human primary myotubes (Gaster *et al.* 2002, Minet & Gaster 2010, Boyle *et al.* 2012, Kase *et al.* 2015). Altogether, our results suggest that the mechanisms responsible for dysregulated skeletal muscle function in PCOS are distinct from those in other metabolic diseases characterized by insulin resistance.

We observed an extensive differential transcriptomic profile between primary myotubes and skeletal muscle, irrespective of the group. We identified in primary myotubes an upregulation of genes related to ECM remodelling, chromatin structure, regulation of protein translation and cellular senescence and a downregulation of genes associated with muscle contraction and multiple mitochondrial functions. Additionally, OXPHOS complex proteins were substantially downregulated in myotubes compared to skeletal muscle tissues, which was accompanied by a significant reduction in mtDNA content. These findings are in line with a previous comparative gene expression profiling study between muscle tissue and myotubes from young healthy females, which showed higher ECM remodelling, cellular senescence and downregulation of genes involved in cellular respiration and OXPHOS in myotubes (Raymond *et al.* 2010). This metabolic adaptation may be explained by the absence of appropriate extracellular signals in the *in vitro* settings (Aas *et al.* 2013), which would lead to reduced mitochondrial content in cultured cells. Therefore, these findings further highlight the contribution of the *in vivo* extracellular milieu in the regulation of gene and protein expression in skeletal muscle of women with PCOS.

In conclusion, our study identified that pathways controlling mitochondrial function and energy metabolism are lowered in skeletal muscle from insulin-resistant women with PCOS, and that these alterations are not preserved in primary myotubes. Our experimental setup comparing transcriptomic profiles of skeletal muscle tissue and derived myotube cultures allowed us to rule out the exclusive contribution of genetic and epigenetic factors in the dysregulation of mitochondrial-associated genes described in PCOS. Our findings open an avenue for the discovery of circulating and paracrine factors present in the extracellular milieu, which could represent targets for the treatment of insulin resistance in PCOS.

## Supplementary Material

Figure S1. RNA-seq pre-processing statistics. x-axis: RNA-seq pre-processing tools used in sequential order as FastQC, STAR and featureCounts (see details in Methods section). y-axis: Number of reads per library. Boxplots show read count statistics of every RNA-seq library (dots). The rows of the table shows library read statistics: n, number of samples/libraries; min/max, minimum/maximum number of reads across libraries; median/mean/iqr, median/mean/interquartile-range of all libraries.

Figure S2. Uncropped immunoblots. (A) Uncropped immunoblots for each of the antibodies (Complex I-V, MRPS7 and MRPL41) with skeletal muscle and myotubes samples from controls and PCOS women. Cropped image shown in Figure 3 is highlighted in red. (B) Representative stain-free total protein content of skeletal muscle and myotubes samples, respectively. (C) Representative immunoblot (OXPHOS Complex II) and stain-free total protein used to calculate correction factors between myotubes (CELLS) and skeletal muscle for each of the antibodies.

Figure S3. MDS plots of RNA-seq data created with batch (participant ID) corrected logCPM values for all skeletal muscle and myotubes. (A) MDS plots indicating age for each sample. (B) MDS plots indicating BMI for each sample. Smaller plots are showing the samples for each unique group in a zoomed-in scale.

## Declaration of interest

The authors declare that there is no conflict of interest that could be perceived as prejudicing the impartiality of the research reported.

## Funding

This work was supported by the National Health and Medical Research Council http://dx.doi.org/10.13039/501100000265 Centre for Research Excellence in PCOS, Australia, and the Novo Nordisk http://dx.doi.org/10.13039/501100004191 Foundation Centre for Basic Metabolic Research, an independent research centre at the University of Copenhagen.
