# Supplementary figures and images for: Non-cell autonomous mechanisms control mitochondrial gene dysregulation in polycystic ovary syndrome
Source: J Mol Endocrinol. 2021 Nov 9;68(1):63–76. doi: 10.1530/JME-21-0212 (PMC8679849; doi:10.1530/JME-21-0212)

Method ■ FastQC ■ STAR ■ featureCounts

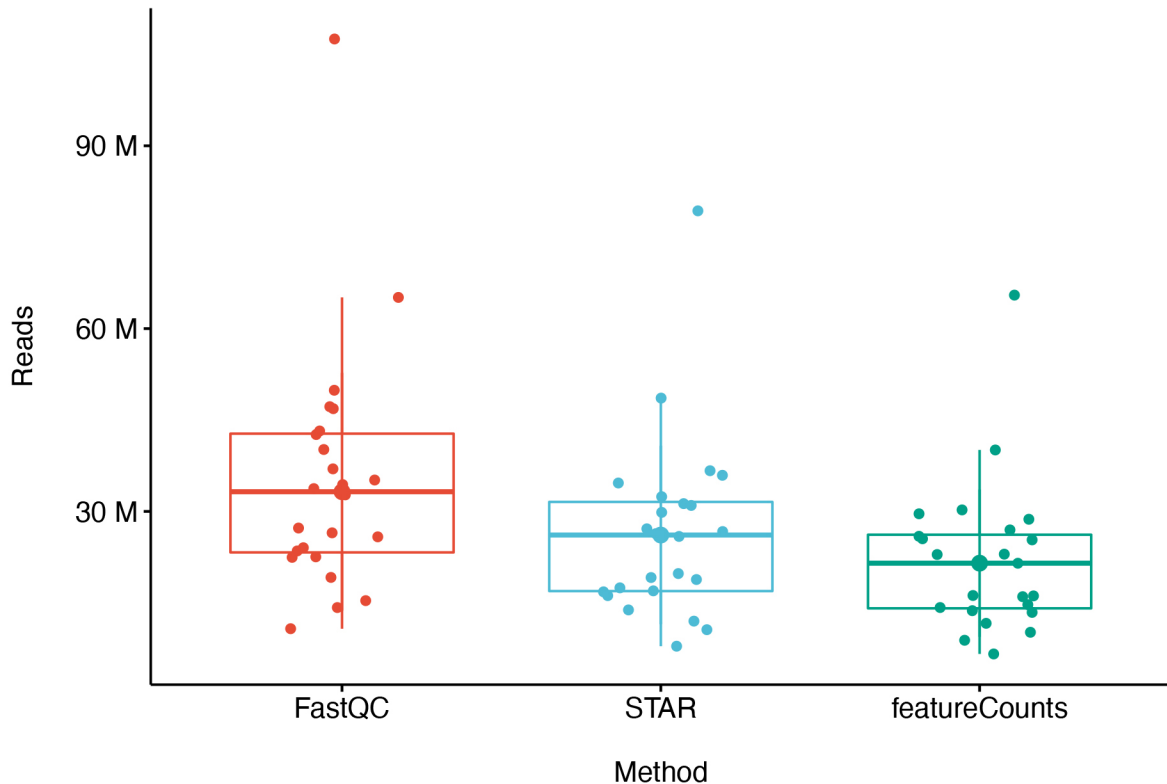

|        |           |          |          |
|--------|-----------|----------|----------|
| n      | 24        | 24       | 24       |
| min    | 10749191  | 7878970  | 6608568  |
| max    | 107527619 | 79324052 | 65493531 |
| median | 33228672  | 26121179 | 21504382 |
| iqr    | 19493104  | 14636958 | 12083569 |
| mean   | 35298244  | 26468228 | 22018509 |

Supplement: Figure S1. RNA-seq pre-processing statistics. x-axis: RNA-seq pre-processing tools used in sequential order as FastQC, STAR and featureCounts (see details in Methods section). y-axis: Number of reads per library. Boxplots show read count statistics of every RNA-seq library (dots). The rows of the ta [file supplementary_figure_1.pdf]

**A**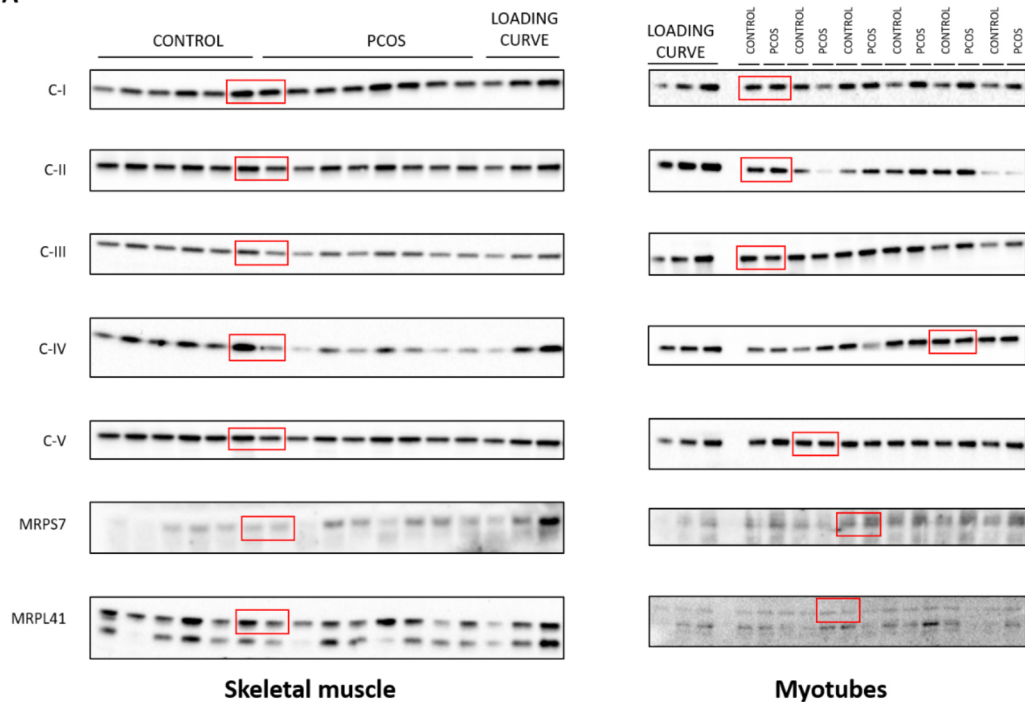**B**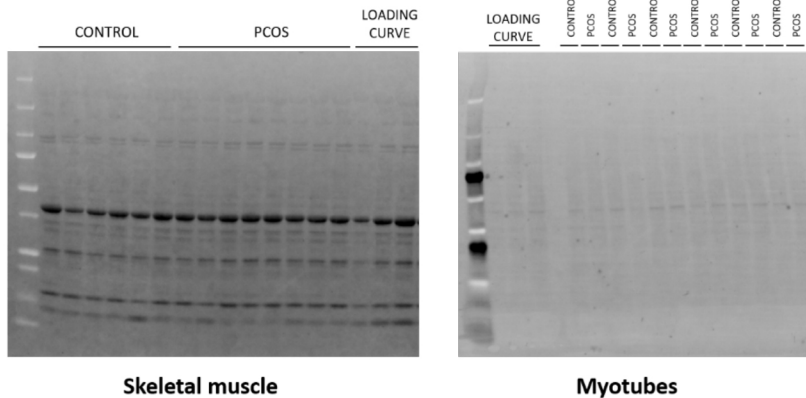**C**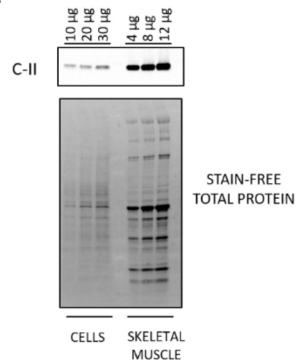

Supplement: Figure S2. Uncropped immunoblots. (A) Uncropped immunoblots for each of the antibodies (Complex I-V, MRPS7 and MRPL41) with skeletal muscle and myotubes samples from controls and PCOS women. Cropped image shown in Figure 3 is highlighted in red. (B) Representative stain-free total protein content of [file supplementary_figure_2.pdf]

A

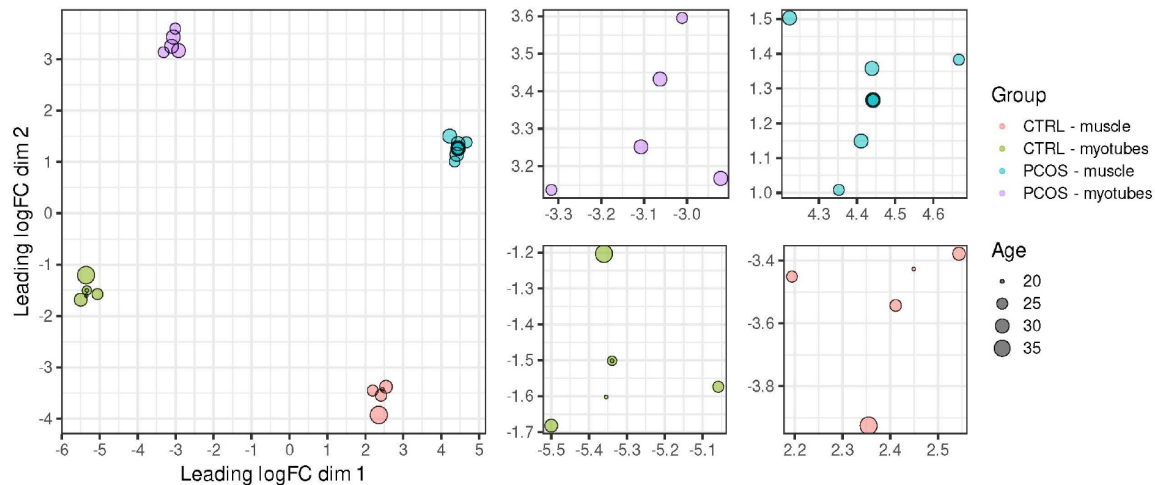

B

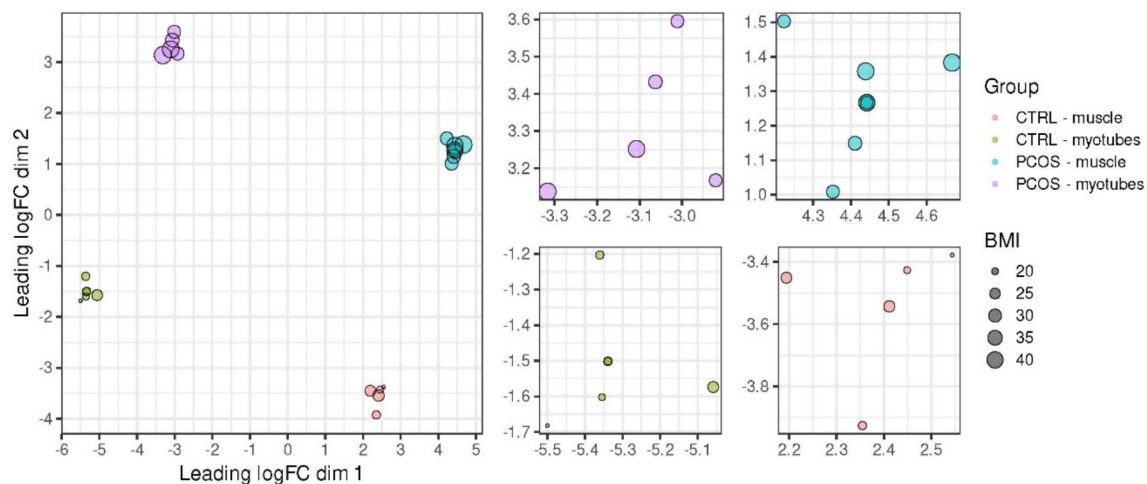

Supplement: Figure S3. MDS plots of RNA-seq data created with batch (participant ID) corrected logCPM values for all skeletal muscle and myotubes. (A) MDS plots indicating age for each sample. (B) MDS plots indicating BMI for each sample. Smaller plots are showing the samples for each unique group in a zoomed-i [file supplementary_figure_3.pdf]
